# Transcriptome profiling of colorectal tumors from patients with sepsis reveals an ethnic basis for viral infection risk and sepsis progression

**DOI:** 10.1038/s41598-022-24489-8

**Published:** 2022-11-30

**Authors:** Natalija Glibetic, Yurii B. Shvetsov, Femke J. Aan, Karolina Peplowska, Brenda Y. Hernandez, Michelle L. Matter

**Affiliations:** 1 University of Hawai’i Cancer Center, Honolulu, HI 96813 USA; 2 Department of Molecular Biosciences and Bioengineering, University of Hawai’i at Mānoa, Honolulu, HI 96822 USA; 3 Tulane University School of Medicine, New Orleans, LA 70112 USA

**Keywords:** Cancer, Computational biology and bioinformatics, Genetics, Diseases, Medical research, Oncology

## Abstract

Mortality from cancer-associated sepsis varies by cancer site and host responses to sepsis are heterogenous. Native Hawaiians have the highest mortality risk from cancer-associated sepsis and colorectal cancer (CRC), even though they demonstrate lower CRC incidence compared to other ethnicities. We conducted a retrospective transcriptomic analysis of CRC tumors and adjacent non-tumor tissue from adult patients of Native Hawaiian and Japanese ethnicity who died from cancer-associated sepsis. We examined differential gene expression in relation to patient survival and sepsis disease etiology. Native Hawaiian CRC patients diagnosed with sepsis had a median survival of 5 (IQR 4–49) months, compared to 117 (IQR 30–146) months for Japanese patients. Transcriptomic analyses identified two distinct sepsis gene signatures classified as early response and late response sepsis genes that were significantly altered in the Native Hawaiian cohort. Analysis of canonical pathways revealed significant up and downregulation in mechanisms of viral exit from host cells (*p* = 4.52E−04) and epithelial junction remodeling (*p* = 4.01E−05). Key genes including elongation initiation factor pathway genes, *GSK3B*, and regulatory associated protein of mTOR (*RPTOR*) genes that protect cells from infection were significantly downregulated in Native Hawaiians. Genes promoting sepsis progression including *CLOCK*,* PPBP* and Rho family GTPASE 2 (*RND2*) were upregulated in Native Hawaiian patients. Our transcriptomic approach advances understanding of sepsis heterogeneity by revealing a role of genetic background and defining patient subgroups with altered early and late biological responses to sepsis. This study is the first to investigate differential gene expression in CRC-associated sepsis patients in relation to ethnicity. Our findings may lead to personalized approaches in stratifying patient mortality risk for sepsis and in the development of effective targeted therapies for sepsis.

## Introduction

Sepsis is a dysregulated immune and vascular leak response to infection by the host that may lead to multiple organ failure (MOF) and is associated with a mortality rate up to 70%^1,2^. Globally, sepsis is a primary or contributing cause of approximately 20% of deaths annually and is the leading cause of death in the intensive care unit (ICU)^3^. Patients with cancer are particularly susceptible to developing sepsis due to depressed immune systems, frequent hospitalizations, surgeries and illness^4^. In the United States alone, 8.5% of total cancer deaths each year are caused by cancer-associated sepsis and approximately 20% of sepsis survivors present with cancer, suggesting a link between sepsis and cancer^3,4^. Retrospective analyses identify cancer as one of the most frequent comorbidities in patients who develop sepsis and cancer patients die from sepsis 2.3 times more frequently than cancer-free patients^5^. Sepsis is significantly associated with increased risk of specific cancers including liver, lung and colon^6^. A transcriptome-based systems biology analysis identified 66 pathways that are enriched in both septic shock and gastrointestinal (GI) cancers^7^. In studies examining adoptees, genetics were found to outweigh environmental effects as sepsis-mediated death risk was five-fold more heritable than cancer-mediated death risk^8^. Moreover, high levels of individual genetic variation in response to sepsis hinders therapeutic treatments and is a key contributing factor to the continued ineffectiveness of clinical trials investigating potential sepsis therapies. Collectively, these findings suggest that genetic variation of a host’s response to infection onset (early response; ERS) and systemic dysregulation (late response; LRS) are key contributors to patient survival outcomes.

Age, sex, underlying chronic conditions, socioeconomic status, environmental factors and ethnicity are all associated risk factors in sepsis etiology and progression^9^. We previously analyzed the Multiethnic Cohort (MEC) for sepsis mortality risk factors and reported that Native Hawaiian cancer patients have a twofold higher mortality risk from sepsis compared to whites, with the hazard ratio (HR) for Japanese and Latinos not significantly different than that of whites^10^. We next performed a retrospective analysis of the MEC to identify cancer associations specific to sepsis and determined colorectal cancer increases the risk of sepsis mortality by 40% compared with non-sepsis mortality^11^. In Hawaii, CRC is highly prevalent as the third most frequently diagnosed and second cause of overall cancer mortality^12^. Enhanced screening and improved therapies over the past decade have significantly decreased CRC-associated mortality rates overall. However, this positive trend has not been observed among Native Hawaiian CRC patients who are generally diagnosed at advanced disease stages and have poor survival outcomes. Importantly, Native Hawaiians have one of the lowest incidences of CRC but highest mortality risk^13,14^. Ethnicity-based genetic variability in CRC-associated sepsis progression and mortality risk may play a role in these observations. Identifying genetic signatures associated with increased risk of CRC-associated sepsis may facilitate more effective preventive measures in cancer patients predisposed to sepsis; however, investigations into sepsis-specific genetic associations have been limited.

Comprehensive transcriptome profiling of CRC tumors has revealed an ethnic basis for pathogenesis^15^. The transcriptome profile of CRC-associated sepsis has not been examined. In this study, we investigated the transcriptome of CRC tumors and adjacent tissue from CRC patients of Native Hawaiian and Japanese ethnicity that died from sepsis with the aim of uncovering interpatient heterogeneity in the transcriptomic response to sepsis. We further investigated ethnic heterogeneity in the transcriptomic response to sepsis by use of pathways, disease phenotypes, predicted upstream regulators and identified context-specific regulatory genetic variants involving gene networks central to sepsis onset and progression.

## Methods

### Study design and study population

The retrospective study population included 15 de-identified deceased colorectal cancer patients of Native Hawaiian or Japanese descent diagnosed in 1990–2007 in Hawaii, aged 35–85+ years with sepsis listed as a cause of death. Sepsis death was defined as a primary or contributing cause of death of A40–A41 according to the International Classification of Diseases (ICD) version 10^16^. De-identified information on patients’ demographic factors, tumor characteristics, and survival was provided by the Hawaii Tumor Registry (HTR), a part of the National Cancer Institute Surveillance, Epidemiology, and End Results (SEER) program. Ethnicity was self-reported with only patients reporting one ethnicity being included in this study.

### Tissue sample selection and RNA extraction

Tumor tissue samples were obtained from a de-identified, retrospective collection of archived, formalin-fixed, paraffin-embedded (FFPE) tumor tissue samples extracted at the time of surgery and maintained by the Hawaii Tumor Registry. All deceased CRC patients satisfying inclusion criteria for whom tissue samples were available were included in this study. Tissue sections were prepared by the Pathology Core of the University of Hawaii Cancer Center using procedures to minimize sample-to-sample contamination and exposure to RNAses including changing blades, cleaning the microtome between samples and by using sterile, RNAse-free disposables. Tissue sections were provided to the Genomics Shared Resource of the UH Cancer Center for RNA isolation and transcriptome profiling.

### Expression profiling by microarray chip assay

Processing of colorectal FFPE RNA samples was performed at the Genomics and Bioinformatics Shared Resource, Cancer Center, University of Hawaii. RNA sample integrity was checked on Agilent 2100 Bioanalyzer using RNA Pico chip. Samples were prepared for microarray hybridization as described in the Thermo Fisher Scientific GeneChip Whole Transcript (WT) Expression manual using the WT Pico Reagent Kit. Double-stranded cDNA was generated from 100 ng of total RNA. Subsequently, cRNA was synthesized using the WT cDNA Synthesis and Amplification Kit (Thermo Fisher Scientific). cRNA was purified and reverse transcribed into single-stranded (ss) DNA. Subsequently a combination of uracil DNA glycosylase (UDG) and apurinic/apyrimidinic endonuclease 1 (APE 1) was used to fragment ssDNA, which was afterwards labeled with biotin (WT Terminal Labeling Kit, Thermo Fisher Scientific). In a rotating chamber, 5.5 μg of fragmented and labeled ss-cDNA were hybridized to the Clariom D Human Array for 16 h at 45 °C. After washing and staining on Affymetrix Fluidics Station FS450 using pre-formulated solutions (Hyb, Wash & Stain Kit, Thermo Fisher Scientific), the hybridized arrays were scanned on the Affymetrix GeneChip Array Scanner 3000-7G. The expression intensity data were extracted from the scanned images and stored as CEL files.

### Statistics, differential gene expression and pathway analysis

Generated CEL files were normalized using the SST-RMA-GENE-FULL algorithm in the Affymetrix Transcriptome Analysis Console (TAC) software (version 4.0.2. for Windows, Waltham, Massachusetts, USA, www.thermofisher.com). Following QC, 6 samples did not pass initial quality control, and one sample is a duplicate to serve as an internal quality control and to assess batch effects. Tumor and adjacent normal tissue were combined for all subsequent analysis because analysis of tumor vs. adjacent normal gene expression among CRC sepsis patients yielded no differentially expressed genes. Differential gene expression was investigated using TAC software. Using a two-sample t-test with a *p* < 0.05 and with adjustment for false discovery rate, we identified 3847 differentially expressed genes with a 1.5-fold difference between Native Hawaiian and Japanese cohorts with sepsis listed as the primary cause of death. Upon removal of pseudogenes, unassigned and noncoding genes from the dataset, 2650 genes remained differentially expressed among the two ethnicities, with 993 genes upregulated and 1656 downregulated. Of those, 2044 genes were analyzed for enriched signaling pathways and functions, predicted upstream regulators and downstream effects with Ingenuity Pathway Analysis (IPA version 01-20-04; Qiagen, Redwood City, CA, USA, www.qiagen.com)^17^. Networks were generated in IPA. Heatmaps were generated with TAC and Graphpad Prism (version 9.0.0 for Mac, San Diego, California USA, www.graphpad.com).

### Ethical approval

This retrospective study was approved by the Institutional Review Board at the University of Hawaii at Mānoa, Honolulu (IRB #11444) and performed in accordance with the UH Human Studies Program guidelines. Prior to data collection, informed consent was obtained from all subjects for the collection of de-identified tissue samples.

## Results

### Transcriptome profiling of Native Hawaiian and Japanese CRC-S patients reveals ethnicity-based genetic variability in CRC-S response and progression

**Figure 1:**
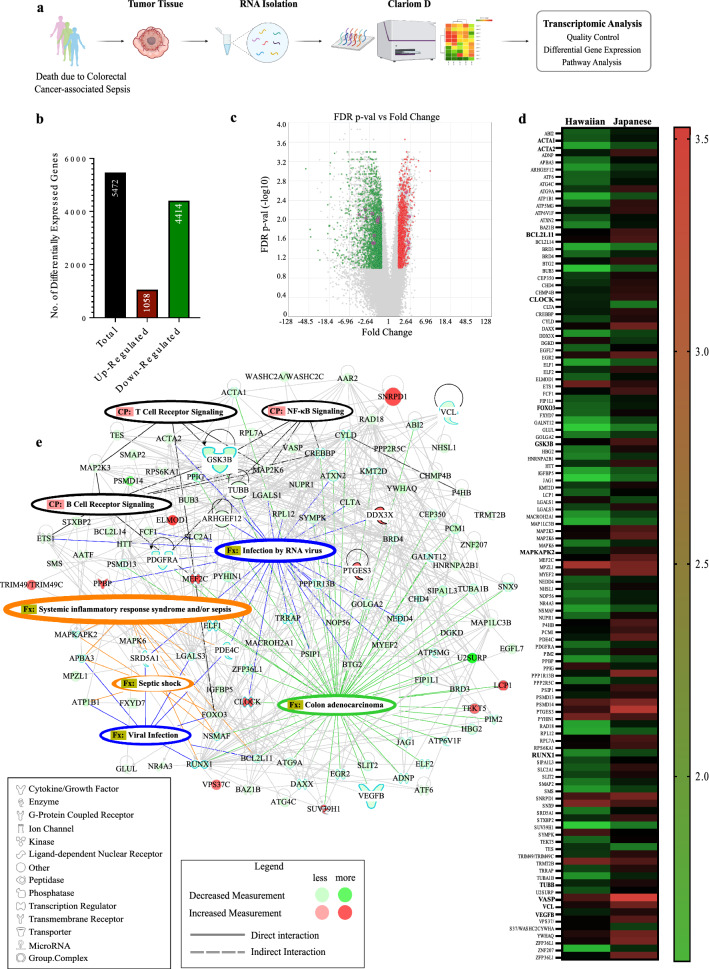
Transcriptome profiling of Native Hawaiian and Japanese colorectal cancer-associated sepsis samples. (**a**) Experimental design. (**b**) Number of total differentially expressed genes in Native Hawaiian samples compared to Japanese. (**c**) Volcano plot of differentially expressed genes for Native Hawaiians vs Japanese (red indicates upregulated genes, and green downregulated genes with a fold change of > 1.5, < − 1.5 and false discovery rate < 0.05). (**d**) Heatmap showing significant up- and down-regulated differentially expressed genes in Native Hawaiians compared to Japanese involved in sepsis, septic shock, viral infection and colon adenocarcinoma. (**e**) Network of most enriched genes from heatmap in (**d**) generated in IPA and their associated canonical pathways (CP). Legend describes log2(FPKM) values.

Native Hawaiian CRC patients diagnosed with sepsis (CRC-S) demonstrated poor survival outcomes after diagnosis that were considerably lower than Japanese CRC-S patients, with a median survival of 5 and 117 months, respectively (Table 1), suggesting a role for ethnicity-based genetic variability in sepsis mortality risk. We therefore investigated interpatient heterogeneity in the transcriptomic response to sepsis in patients from Native Hawaiian and Japanese ethnic backgrounds (Fig. 1a). Patient and tumor characteristics of study participants are summarized in Table 2. We performed analysis with baseline and tumor characteristics by ethnicity and found that there is no significant correlation of ethnicity with other phenotypes as supported by the frequency counts shown in Table 2. Principal component analysis of global gene expression for CRC tumors revealed two distinct clusters that correlated only with ethnicity, and not with any other factors including sex, age or disease stage (Supplemental Figure S1A–D). Unsupervised hierarchical cluster analysis of global gene expression for CRC tumors revealed distinct expression patterns for Native Hawaiian samples compared to Japanese samples (Supplemental Figure S2). We identified 5472 significant differentially expressed genes between cohorts (FC > 1.5, < − 1.5; FDR < 0.05) with 1058 (19.3%) upregulated and 4414 (80.7%) downregulated in Native Hawaiian CRC-S patients compared to Japanese CRC-S patients (Fig. 1b,c; Supplemental Figure S3). In agreement with Davenport et al.^18^, we saw no gene expression differences in inflammatory cytokines interleukin 1 beta (*IL1B*)*,* interleukin 6 (*IL6*) and 10 (*IL10*), tumor necrosis factor (*TNF*), transforming growth factor beta (*TGFB*)*,* or interferon alpha (*IFNA*), beta (*IFNB*) and gamma (*IFNG*) genes across septic patients. However, pathway analysis of differentially expressed genes identified functional differences related to viral infection, B cell and T cell signaling, systemic inflammatory response/sepsis and septic shock (Fig. 1d,e). Key genes controlling viral infection, systemic inflammatory responses, sepsis, and septic shock were differentially expressed in the Native Hawaiian cohort including *RUNX1* (FC = − 2.11, *p* = 0.001), *BCL2L11* (FC = − 2.27, *p* = 0.002), *FOXO3* (FC = − 3.27, *p* = 1.00E−04) and *MAPKAPK2* (FC = − 3.2, *p* = 2.00E−04).

**Table 1:** Survival among study participants, by ethnicity, stage, and treatment status.

Characteristic	Survival time (months)	
	Median (IQR)	Mean (std)
All participants	49 (13–146)	86.6 (82.7)
**Sex**		
Females	97.5 (30–187)	113.2 (101.4)
Males	30 (13–129)	68.9 (68.3)
**Ethnicity**		
**Hawaiians**	**5 (4–49)**	**64.4 (112.2)**
**Japanese**	**117 (30–146)**	**97.7 (68.1)**
**Stage**		
Localized	146 (49–172)	130 (78.4)
Regional	67 (4.5–158)	81.3 (91.7)
Distant	16 (7.5–24.5)	16 (11.7)
**Treatment**		
Chemotherapy	30 (5–172)	74.6 (79.5)
No chemotherapy	89 (21.5–146)	97.1 (89.4)
Radiation treatment	105 (5–129)	83.0 (75.6)
No radiation treatment	39.5 (19–146)	88.4 (90.0)

Overall Survival Time by Ethnicity are in [bold].

**Table 2:** Baseline and tumor characteristics of study participants.

Characteristic	All participants	Hawaiian (N = 5)	Japanese (N = 10)
	N (%)	N (%)	N (%)
**Sex**			
Female	6 (40.0%)	3 (60.0%)	3 (30.0%)
Male	9 (60.0%)	2 (40.0%)	7 (70.0%)
**Age group**			
< 65 years	5 (33.3%)	3 (60.0%)	2 (20.0%)
65+ years	10 (66.7%)	2 (40.0%)	8 (80.0%)
**Cancer stage at diagnosis**			
Localized	7 (46.7%)	2 (40.0%)	5 (50.0%)
Regional	4 (26.7%)	2 (40.0%)	2 (20.0%)
Distant	4 (26.7%)	1 (20.0%)	3 (30.0%)
**Cancer grade at diagnosis**			
Moderately differentiated	10 (66.7%)	3 (60.0%)	7 (70.0%)
Poorly differentiated	3 (20.0%)	2 (40.0%)	1 (10.0%)
Unknown	2 (13.3%)	0 (13.3%)	2 (20.0%)
**Chemotherapy**			
Yes	7 (46.7%)	2 (40.0%)	5 (50.0%)
No/unknown	8 (53.3%)	3 (60.0%)	5 (50.0%)
**Radiation treatment**			
Yes	5 (33.3%)	2 (40.0%)	3 (30.0%)
No/unknown	10 (66.7%)	3 (60.0%)	7 (70.0%)

**Figure 2:**
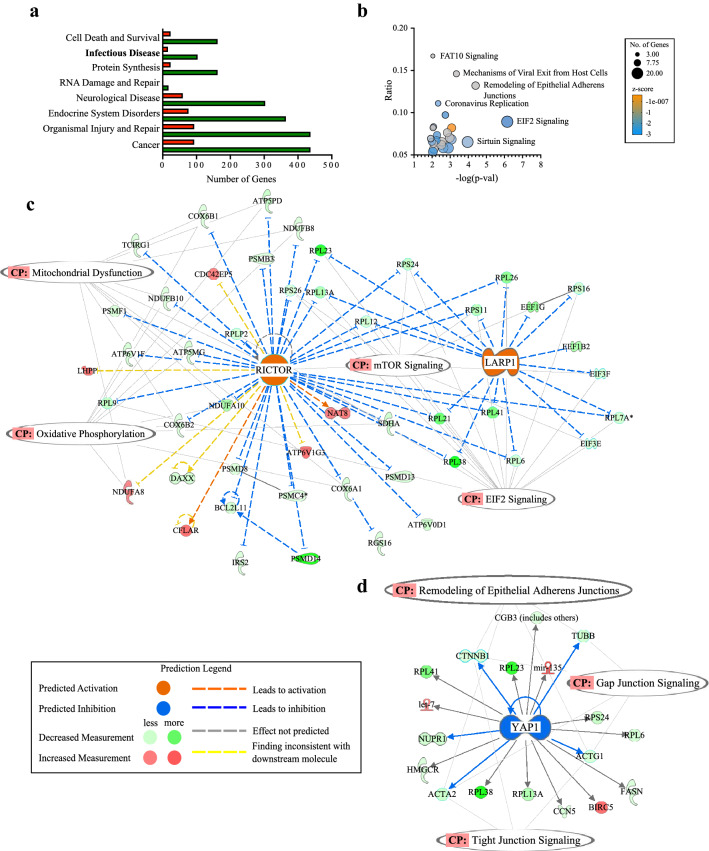
Top disease phenotypes, canonical pathways and predicted upstream regulators of differentially expressed genes in Native Hawaiians when compared to Japanese. (**a**) Number of up- and downregulated genes in most enriched disease phenotypes. Green bars are downregulated genes, red bars are upregulated genes. (**b**) Bubble plot indicating the top canonical pathways, where bubble size corresponds to number of genes enriched for corresponding pathway and color indicates z score. Orange bubbles indicate predicted activation and a positive z score, blue bubbles indicate predicted inhibition and a negative z score. Grey bubbles indicate no z score or a z score of 0. (**c**) Network of genes affected by predicted upstream regulators Lh, LARP1, and RICTOR, and their associated canonical pathways (CP). (**d**) Network of genes affected by YAP1 and associated CPs.

Genes involved in viral infection, sepsis and septic shock displayed high levels of heterogeneity with significant differential expression in the Native Hawaiian cohort compared to the Japanese group (Fig. 1E). Top disease phenotypes with significantly altered gene expression in Native Hawaiians CRC-S patients include cancer (*p* = 2.97E−16–2.70E−04), organismal injury and abnormalities (*p* = 2.97E−16–1.52E−02), protein synthesis (*p* = 3.18E−09–1.11E−02), infectious disease (*p* = 3.41E−09–1.32E−02) and cell death and survival (*p* = 2.31E−07–1.52E−02; Fig. 2a) with significant overall downregulation in genes that affect these phenotypes. Analysis of canonical pathways revealed significant gene alterations in mechanisms of viral exit from host cells (*p* = 4.52E−04), coronavirus replication (*p* = 0.005), translation initiation factor *EIF2* (*p* = 7.09E−07) that promotes translation of viral proteins in host cells, and epithelial junction remodeling (*p* = 4.01E−05; Fig. 2b). The most significant predicted upstream regulators include inflammation response genes (*LAPR1*; FC = − 3.25, *p* = 1.74E−09), RPTOR independent companion of MTOR complex 2 (*RICTOR*; *p* = 1.51E−07; Fig. 2c) and *YAP1* (*p* = 1.01E−06; Fig. 2d). The transcription regulator *MYCN* (*p* = 6.08E−08; Supplemental Figure S4) was also differentially expressed and is primarily involved in cancer specific signaling.

### Early response sepsis (ERS) genes involved in viral infection and inflammation are differentially expressed in Native Hawaiian compared to Japanese CRC-S patients

**Figure 3:**
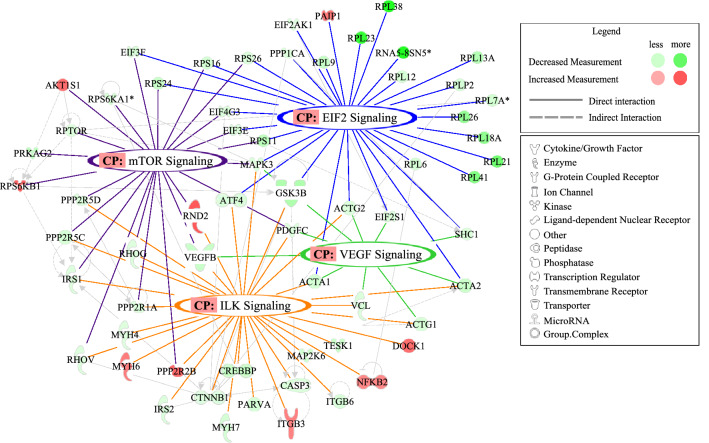
Differentially expressed genes between Native Hawaiians and Japanese reveal Early Response Sepsis (ERS) genes involved in viral infection. Network of top canonical pathways EIF2, mTOR, ILK and VEGF and interactions between associated genes identified by IPA. *CP* canonical pathway.

Further pathway analysis of differentially expressed genes identified distinct early response sepsis gene signatures that regulate infection and inflammation and late response sepsis (LRS) genes involved in adherens junction signaling and vascular leak, a component of sepsis progression. ERS signaling pathways including EIF2 (*p* = 6.59E−07, z-score: − 3.30), integrin-linked kinase (ILK; *p* = 1.23E−05, z-score: − 1.62), and mTOR (*p* = 0.042; z-score: 0) were among the most significantly affected canonical pathways in Native Hawaiian CRC-S patients (Fig. 3). A total of 31 genes associated with EIF2 signaling, which was predicted to be inhibited, were identified with ribosomal protein L38 (*RPL38;* FC = − 16.9, *p* = 3.09E−05), 5.8S ribosomal N5 (*RNA5*; FC = − 12.7, *p* = 0.002), *RPL23* (FC = − 11.2, *p* = 0.004) being the most downregulated and poly(A) binding protein interacting protein 1 (*PAIP1;* FC = 1.74, *p* = 6.00E−04) being the only upregulated gene (Fig. 3). Moreover, *RPL38* was among the topmost downregulated genes in Native Hawaiian samples compared to those from Japanese patients (Supplemental Figure S5). *GSK3B* (FC = − 4.38, *p* = 2.00E−04), *MAPK3* (FC = − 1.92, *p* = 0.002) and *ACTA1* (FC = − 2.30, *p* = 6.00E−04) were downregulated in the Native Hawaiian cohort compared to the Japanese cohort and these genes overlap with ILK, mTOR and VEGF canonical signaling pathways. Among the 32 genes associated with ILK signaling, *GSK3B*, *MAPK3*, and *VEGFB* (FC = − 2.94, *p* = 1.92E−05) were among the most downregulated genes, while *RND2* (FC = 2.16, *p* = 1.99E−05), *PPP2R2B* (FC = 1.96, *p* = 3.03E−05), and dedicator of cytokinesis 1 (*DOCK1*; FC = 1.89, *p* = 8.77E−05) were among the most upregulated genes. Moreover, *ACTA1*, *ACTA2* (FC = − 2.77, *p* = 3.50E−03) and *VEGFB* cross talk with numerous pathways and signal on the VEGF pathway, which also includes *MAPK3,* vinculin (*VCL*; FC = − 2.01, *p* = 6.00E−04), and *SHC1* (FC = − 1.67, *p* = 0.004). Collectively, these findings support ethnicity-based gene regulation changes in response to sepsis early response genes.

### Late response sepsis (LRS) genes involved in permeability regulation are differentially expressed in Native Hawaiian compared to Japanese CRC-S patients

**Figure 4:**
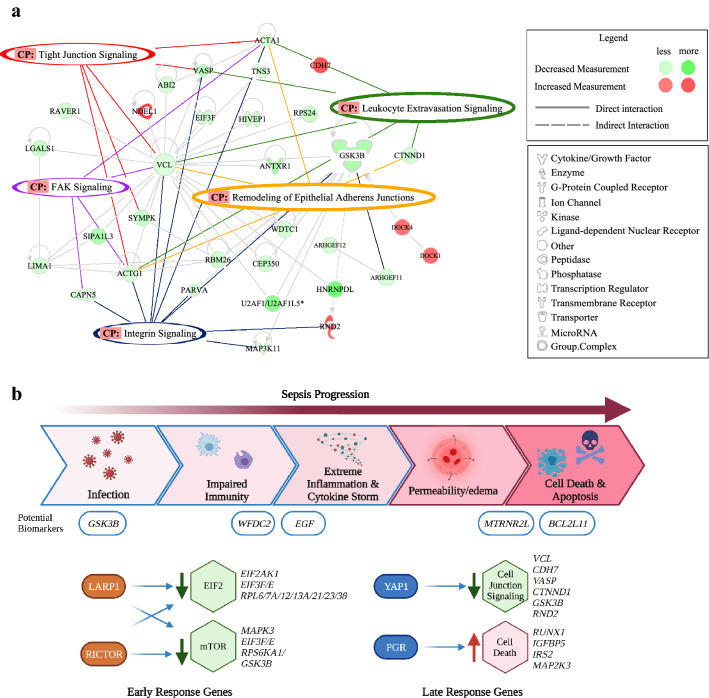
Differentially expressed genes between Native Hawaiians and Japanese reveal Late Response Sepsis (LRS) genes involved in cell–cell junction regulation and barrier permeability. (**a**) Differentially expressed network indicating the connections and interacting partners of permeability-regulating junction canonical pathways (tight junction signaling, leukocyte extravasation signaling, remodeling of epithelial adherens junctions, FAK signaling, and integrin signaling). (**b**) Schematic of sepsis progression steps with listed potential biomarkers and associated ERS and LRS genes grouped by their predicted upstream regulators and affected pathways.

Epithelial junction remodeling is a key mechanism of cancer progression^19^ yet also essential in the pathophysiological progression from sepsis to septic shock^20^. These LRS regulated genes control key components of late-stage sepsis including initiation of vascular and intestinal leak. Increased vascular leak further exacerbates MOF by inducing tissue edema^20^. LRS gene signatures regulating epithelial adherens junction formation and vascular barrier integrity were significantly downregulated in the Native Hawaiian cohort when compared to the Japanese cohort (*p* = 4.01E−05). LRS genes including *VCL*, a cytoskeletal remodeling protein involved in junctional remodeling, tubulin beta (*TUBB6*; FC = − 2.40, *p* = 0.002), ras-related protein Rab-5B (*RAS5B*; FC = − 2.08, *p* = 0.005*),* tubulin alpha 1b (*TUBA1B*; FC = − 2.02, *p* = 0.003), actin alpha 1 (*ACTA1*; FC = − 2.30, *p* = 6.00E−04), catenin delta 1 (*CTNND1*; FC = − 2.27, *p* = 0.001), tubulin alpha-1C chain (*TUBA1C*; FC = 2.43, *p* = 0.002), actin gamma 1 (*ACTG1*; FC = − 2.09, *p* = 0.004), protein kinase AMP-activated non-catalytic subunit gamma 2 (*PRKAG2*; FC = − 3.33, *p* = 4.00E−04), and vesicle transport through interaction with t-SNAREs 1A (*VTI1A*; FC = − 2.16, *p* = 0.001) are all downregulated in Native Hawaiian patient tumors compared to Japanese (Fig. 4a). IPA analysis of candidate biomarkers identified both ERS and LRS genes including *GSK3B, WDC2*, *FOXO3*, *LDH*, *MTRNR2L* and *BCL2L11* (Supplemental Table S1; Fig. 4b). Collectively, such alterations point to a genetic signature whereby genes controlling vascular and intestinal permeability and epithelial junction remodeling in healthy cells are significantly downregulated in Native Hawaiian CRC-S patients compared to Japanese CRC-S patients.

## Discussion

We propose that individual transcriptomic signatures, which define ethnic variation in the host response to sepsis will help develop a precision medicine-based approach for cancer-associated sepsis management, improve the basis of selection of patients for inclusion in clinical trials, identify new drug targets and allow for more rapid preventive measures in cancer patients predisposed to sepsis. Our data suggests the host response is, in part, regulated by an ethnic gene signature response in viral infection, sepsis onset and progression. We demonstrate that Native Hawaiian CRC-S patients have reduced survival outcomes that may be explained, in part, by key differences in specific sepsis gene signatures compared with Japanese CRC-S patients. Moreover, transcriptional differences between Native Hawaiians and Japanese CRC-S patients suggest early and late responses are significantly altered in sepsis progression, which may be reflected in the potential biomarkers we identified such as *GSK3B, WFDC2* and *MTRNR2L1* (Fig. 4b).

While significant changes in CRC specific cancer genes were expected as the role of ethnic heterogeneity has been more fully studied in cancer^21,22^, this is the first analysis on differential expression in gene signatures of CRC-associated sepsis and ethnic heterogeneity. Native Hawaiians demonstrated significantly altered gene expression relating to tumorigenesis and cell death but also to infectious disease, systemic inflammation/sepsis, and septic shock. Considering Native Hawaiian CRC-S patients have a dramatically poor prognosis with early mortality due to sepsis, we focused on the differential gene signature alterations involved in the host’s early and late responses to sepsis (Fig. 4b). To this end, we examined gene changes in host response to infection and vascular permeability not limited to tumor or normal tissue and therefore included both tissues in our analysis. Viral infection, inflammation and vascular permeability pathways were significantly altered in the Native Hawaiian CRC-S cohort. ERS genes include host responses to viral infection and subsequent inflammation whereas LRS gene changes include adherens junctions, permeability, and vascular barrier integrity (Fig. 4b).

Pathway analysis identified genetic variants associated with the expression of genes involved in key immune and metabolic responses to infection (Fig. 3). One ERS pathway downregulated in the Native Hawaiian sepsis group is eIF2α, which acts as a sensor of virus load where dsRNA or viral proteins generated by viral proliferation activate eIF2α kinases inducing phosphorylation that abrogates protein synthesis in the host cell. This limits the ability of viruses to proliferate^23^. The gene changes in eIF2α and eIF3 kinases observed in the Native Hawaiian cohort may allow for increased virus proliferation as this host protective response pathway is significantly downregulated.

We identified significant changes in mTOR pathway genes including *RHOG, EIF3,* and *LARP1* (Fig. 3). IPA analysis predicted the RICTOR-mTOR signaling pathway to be activated in Native Hawaiians CRC-S patients compared to Japanese CRC-S patients. Activation of RICTOR leads to inhibition of downstream genes including *RPL, BCL211* and proteasomal subunits *PSMD13/14* among others (Fig. 2c). RPTOR independent companion of MTOR complex 2 (*RICTOR*) is important in inflammation signaling pathways as is the downstream transcription regulator YAP1. The RICTOR/mTORC2 signaling pathway is protective against LPS inflammation by inhibiting YAP1 degradation and NFκB translocation to the nucleus^24^; a pathway that was specifically downregulated in Native Hawaiians with CRC-associated sepsis (Fig. 2d). The downstream *GSK3B* gene was significantly downregulated in Native Hawaiians CRC-S patients and this gene plays a role in sepsis onset and progression. Activation of a GSK-3*β*/mTOR pathway is protective in a sepsis preclinical animal model^25^ and dexmedetomidine treatment is also protective in LPS-induced acute lung injury in a rat preclinical model via activation of a PI3K/Akt/mTOR pathway^26^. The observed downregulation of the *GSK3B* gene and its effects on numerous pathways highlighted in our IPA analyses point to its role as a potential gate keeper of sepsis progression. RICTOR-mTOR signaling impacts other pathways including mitochondrial function and oxidative phosphorylation. OX-phos and mitochondrial function are dysregulated during sepsis, which may lead to MOF. Canonical pathway analysis points to downregulation of mitochondrial function and changes in oxidative phosphorylation in Native Hawaiian CRC-S patients compared to Japanese CRC-S patients. These findings suggest inhibition of the GSK-3*β*/mTOR pathway is detrimental to the host’s response to sepsis and is in keeping with our data that downregulation of this sepsis gene signature correlates with poor prognosis in Native Hawaiians CRC patients presenting with sepsis.

IPA pathway analysis identified genetic variants associated with the expression of genes involved in late responses to sepsis including alterations in adherens junctions, intestinal and vascular permeability (Fig. 4a). Changes in adherens junction proteins and increased permeability together promote vascular leak in sepsis that leads to tissue edema, MOF, septic shock, and death. Downregulated LRS genes include vasodilator-stimulated phosphoprotein (*VASP*)*,* vinculin *(VCL)*, and F-actin. VASP protein expression is involved in cytoskeletal rearrangements, cell movement and cell–cell junctions. Indeed, abrogating VASP expression induces edema in mice^27^. Vinculin protein associates with focal adhesion and adherens junction proteins to regulate endothelial barrier function and an association of F-actin with ZO-1 is essential for controlling vascular permeability.

While we observed the most significant gene changes led to downregulation of genes and pathways, we identified several upregulated genes in the Native Hawaiian sepsis cohort including Cadherin 7*, CLOCK* and *RND2*. Upregulation of Cadherin occurs during sepsis and has been identified as a potential biomarker in sepsis endothelial dysfunction^28^. Circadian clock genes are essential in regulating inflammation, cell cycle and tissue repair. Mice with the circadian gene *CLOCK* deleted demonstrate increased survival outcomes compared with wild type mice in a polymicrobial sepsis preclinical model^29^. The upregulation of the *CLOCK* gene in the Native Hawaiian CRC-sepsis cohort suggests that this may be a potential negative prognosis marker. The RND family of RHO GTPases 2 (*RND2*) is a negative regulator of the actin cytoskeleton^30^ and we identified upregulation of *RND2* in Native Hawaiians with sepsis, which may alter vascular barrier integrity to induce vascular leak.

There are limitations to this study. The small sample size of the present study limited our ability to examine transcriptome differences in smaller strata and in relation to other factors, such as behavioral, socioeconomic and environmental factors. A larger sample size would be required for sufficient power to identify moderate genetic signature changes. Moreover, access to external validation sets would strengthen our findings; however at this time there are none available. In the future we hope to develop large validation sets of CRC patient tumors with and without sepsis from various ethnicities for further comparison, which would enhance validation of researchers findings. This is the first study to analyze the CRC-patient tumor transcriptome of different ethnicities with and without sepsis. We recognize that further research is required to understand the cellular and tissue specificities of our data. It will be important to establish whether the genetic changes identified can be found in other ethnicities and in patients with sepsis from different causes. Evaluating the transcriptome of patients in the clinic might be useful for identifying cancer patients predisposed to sepsis and those who may benefit from adjunctive corticosteroids or potential pathway inhibitors. Proteomics would further validate our findings.

There is an urgent medical need to identify host responses to sepsis due to the high mortality rate and lack of targeted therapies. Currently, there are no accurate means to identify which patients are predisposed to sepsis. Our analysis begins to expose the ethnicity-mediated genetic changes in cancer patients with sepsis and provide insight into ethnic diversity in genetic signatures in response to severe illness. These novel genetic signatures could allow for the identification of high-risk patients, development of predictive biomarkers and targeted therapies to block sepsis progression.

## Supplementary Information

Supplementary Information.

## Data Availability

Transcriptomic data were deposited in the NCBI Gene Expression Omnibus database (accession no. GSE198103). Additional requests should be made to mmatter@tulane.edu.
