# Supplementary material for: Transcriptome profiling of colorectal tumors from patients with sepsis reveals an ethnic basis for viral infection risk and sepsis progression
Source: Sci Rep. 2022 Nov 30;12:20646. doi: 10.1038/s41598-022-24489-8 (PMC9709755; doi:10.1038/s41598-022-24489-8)
Supplement: Supplementary file 1 — Supplementary Information. [file 41598_2022_24489_MOESM1_ESM.pdf]

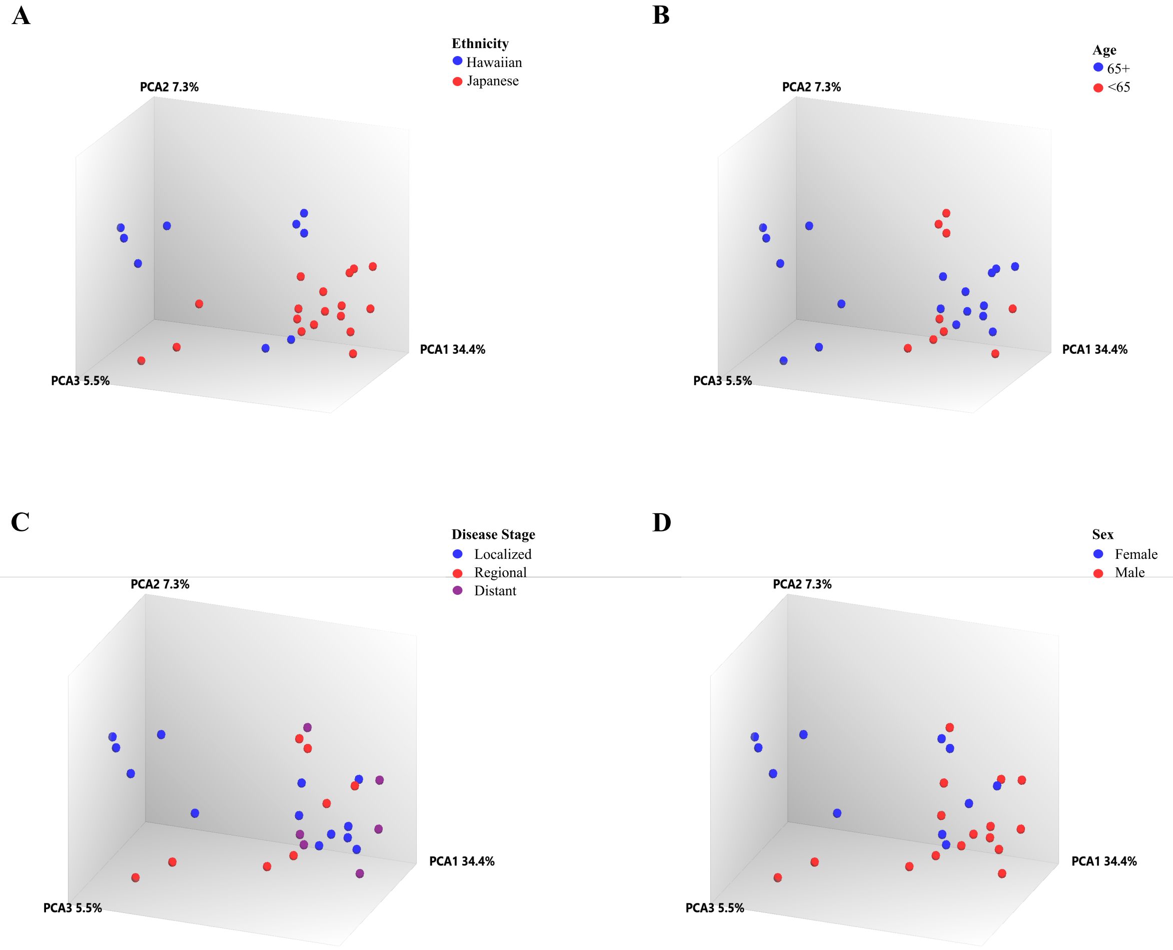

Supplemental Figure S1. Gene expression profiles differ in Native Hawaiian and Japanese colorectal cancer tumors from patients that died from sepsis. A) Principal component analysis (PCA) of total gene expression profiles generated with Transcriptome Analysis Console (TAC; version 4.0.2.) colored by **A)** ethnicity (blue=Hawaiian, red=Japanese), **B)** age (blue=65+, red=<65), **C)** disease stage (blue=localized, red=regional, purple=distant), and **D)** sex (blue=female, red=male) with the proportion of variance explained by each component.

A

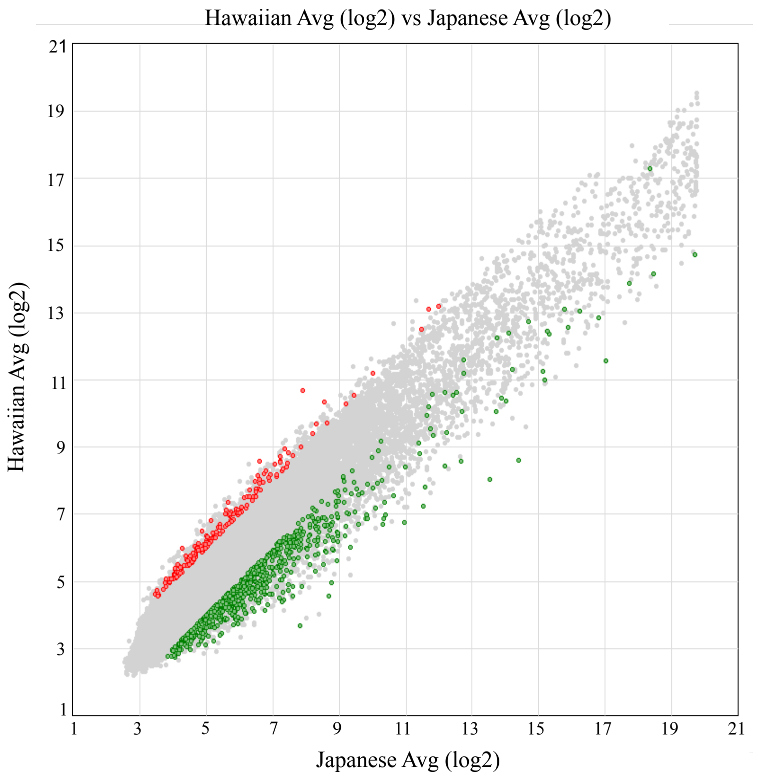

Supplemental Figure S2. Differential gene expression of colorectal tumors from Native Hawaiians patients with sepsis when compared to Japanese patients with sepsis. A) Scatter plots generated with TAC software (v 4.0.2.) showing filtered up- (red; FC>1.5, FDR<0.05) and downregulated (green; FC<-1.5, FDR<0.05) genes in Native Hawaiians when compared to Japanese.

A

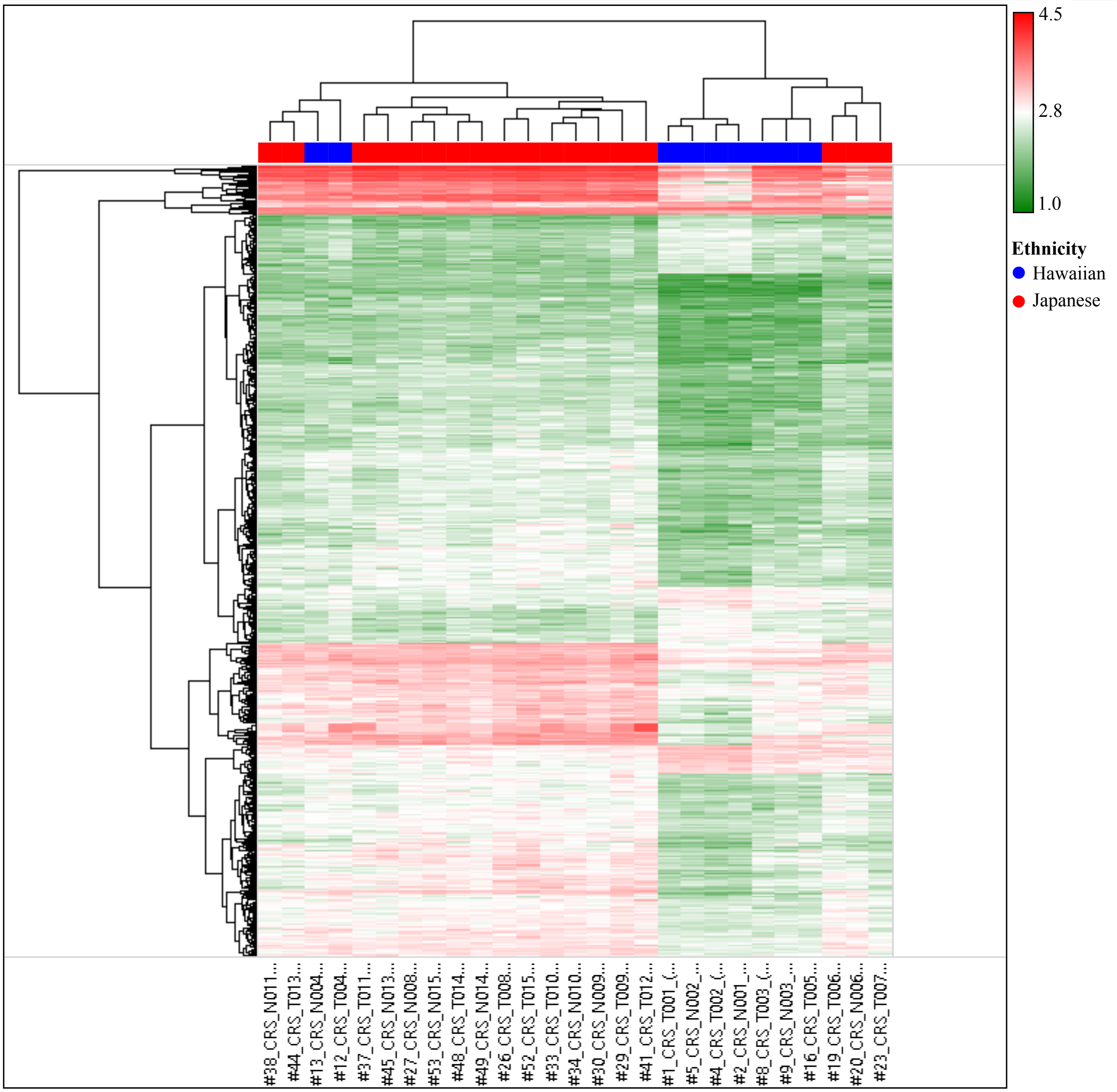

Supplemental Figure S3. Hierarchical clustering reveals distinct gene expression in colorectal tumors with sepsis based on ethnicity. A) Unsupervised hierarchical cluster analysis of gene profiles generated by TAC (v4.0.2.) separates colorectal tumor samples by ethnicity. Ethnicity is labeled by color (blue=Hawaiian, n=9; red=Japanese, n=18). Genes display upregulation (red; FC>1.5, FDR<0.05) and downregulation (green; FC<-1.5, FDR<0.05).

A

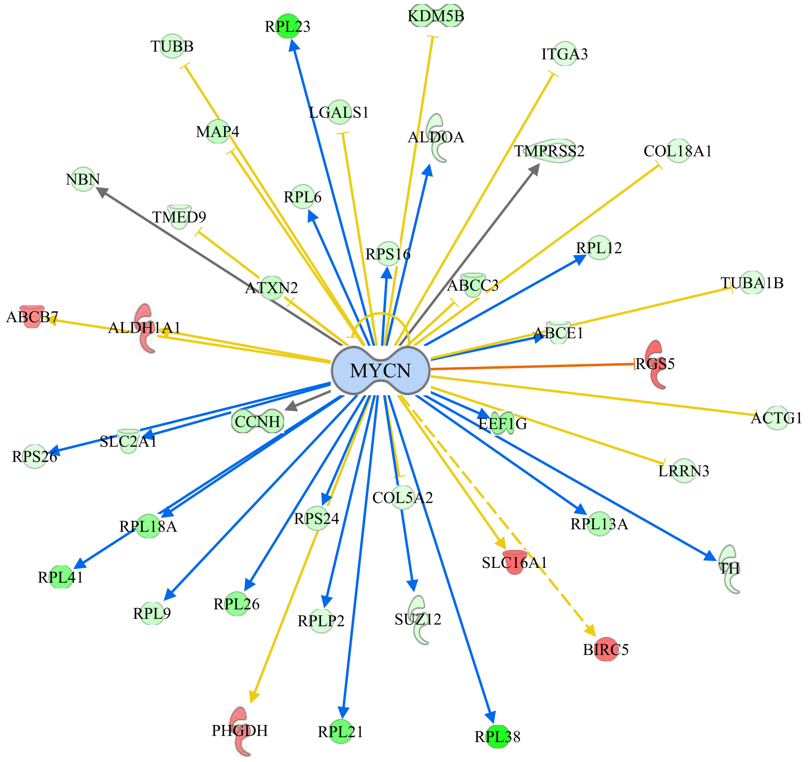

Supplemental Figure S4. MYCN signaling is predicted to be inhibited in Native Hawaiian colorectal tumors with sepsis when compared to those from Japanese patients. A) Network of genes affected by predicted upstream regulators MYCN generated by Ingenuity Pathway Analysis. Green genes are downregulated, red genes are upregulated. Blue arrows predict inhibition, yellow arrows indicate that the finding is inconsistent with the state of the downstream molecule. Full arrows indicate direct interaction, dotted lines indicate indirect interaction.

A

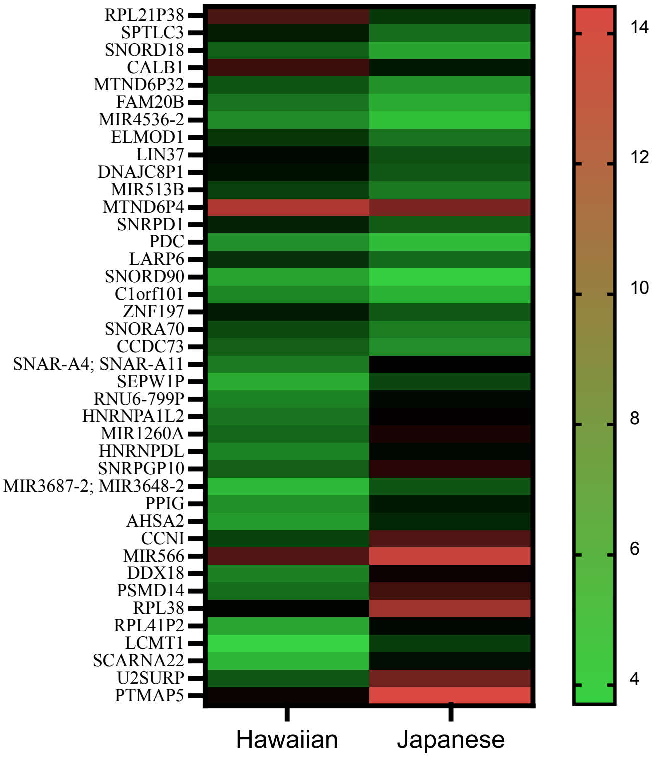

Supplemental Figure S5. Top upregulated and downregulated genes in Native Hawaiian colorectal tumors with sepsis. A) Heatmap of top 20 upregulated (FC>2, FDR<0.05) and downregulated (FC<-2, FDR<0.05) genes in Native Hawaiian colorectal tumors with sepsis when compared to those from Japanese patients. Legend indicates log2(FPKM) values. Figure was prepared using GraphPad Prism 9.

**Supplemental Table S1. Candidate biomarkers identified by IPA biomarker filter analysis**				
**Symbol**	**Gene Name**	**Location**	**Drug(s)**	**Biomarker Application**
ACTA2	Actin alpha 2, smooth muscle	Cytoplasm	N/A	Efficacy
BCL2L11	Bcl2 like 11	Cytoplasm	N/A	Efficacy
CASP3	Caspase 3	Cytoplasm	caspase 3 inhibitor	Diagnosis, disease progression, efficacy, unspecified application
FOXO3	Forkhead box O3	Nucleus	N/A	Efficacy
GSK3B	Glycogen synthase kinase 3 beta	Nucleus	glycogen synthase kinase-3beta inhibitor, enzastaurin	Efficacy
LDHC	Lactate dehydrogenase C	Cytoplasm	N/A	Diagnosis
MTRNR2L1	MT-RNR2 like 1	Other	N/A	N/A
IRS1	Insulin receptor substrate 1	Cytoplasm	NT219	Efficacy
LGALS1	Galectin 1	Extracellular space	OTX008	Diagnosis, prognosis
LGALS3	Galectin 3	Extracellular space	GCS-100, GR-MD-02	Diagnosis, unspecified application
PDGFRA	Platelet derived growth factor receptor alpha	Plasma membrane	decitabine/imatinib, ripretinib, nintedanib, regorafenib/sunitinib, imatinib/sirolimus, dasatinib, imatinib/sorafenib, avapritinib, filgrastim/imatinib, midostaurin, lapatinib/pazopanib, imatinib/inotuzumab ozogamicin, lenvatinib, olaratumab, imatinib/sunitinib, sunitinib, axitinib, 5-azacytidine/midostaurin, regorafenib, pazopanib, telatinib, imatinib, imatinib/rituximab, crenolanib, becaplermin, amuvatinib, imatinib/nilotinib, imatinib/peginterferon alfa-2a, erdafitinib, lenvatinib/pembrolizumab, crizotinib/pazopanib, blinatumomab/imatinib, bosutinib/imatinib, docetaxel/nintedanib, everolimus/lenvatinib	Efficacy, prognosis, safety, unspecified application
TUBB	Tubulin beta class I	Cytoplasm	ixabepilone/rifampin, ixabepilone	Efficacy
VCL	Vinculin	Plasma membrane	N/A	Unspecified application
VEGFB	Vascular endothelial growth factor B	Extracellular space	aflibercept, aflibercept/irinotecan, aflibercept/bevacizumab	Efficacy, prognosis
WFDC2	WAP four-disulfide core domain 2	Extracellular space	N/A	Diagnosis, unspecified application
